# Does Muscle Pain Induce Alterations in the Pelvic Floor Motor Unit Activity Properties in Interstitial Cystitis/Bladder Pain Syndrome? A High-Density sEMG-Based Study

**DOI:** 10.3390/s24237417

**Published:** 2024-11-21

**Authors:** Monica Albaladejo-Belmonte, Michael Houston, Nicholas Dias, Theresa Spitznagle, Henry Lai, Yingchun Zhang, Javier Garcia-Casado

**Affiliations:** 1 Centro de Investigación e Innovación en Bioingeniería (CI2B), Universitat Politècnica de València, Camino de Vera s/n, 46022 Valencia, Spain; moalbel@ci2b.upv.es; 2 Department of Biomedical Engineering, University of Houston, Houston, TX 77204, USA; mjhouston2@uh.edu (M.H.); nicholascdias@gmail.com (N.D.); 3 Program in Physical Therapy, Washington University School of Medicine, St. Louis, MO 63108, USA; spitznaglet@wustl.edu; 4 Division of Urologic Surgery, Departments of Surgery and Anesthesiology, Washington University School of Medicine, St. Louis, MO 63110, USA; laih@wustl.edu; 5 Department of Biomedical Engineering, University of Miami, Coral Gables, FL 33146, USA; 6 Desai Sethi Urology Institute, University of Miami, 1120 NW 14th Street, Miami, FL 33136, USA; 7 Miami Project to Cure Paralysis, University of Miami, 1095 NW 14th Terrace #48, Miami, FL 33136, USA

**Keywords:** coherence, high-density surface electromyography, interstitial cystitis/bladder pain syndrome, motor unit, pelvic floor muscles

## Abstract

Several studies have shown interstitial cystitis/bladder pain syndrome (IC/BPS), a chronic condition that poses challenges in both diagnosis and treatment, is associated with painful pelvic floor muscles (PFM) and altered neural drive to these muscles. However, its pathophysiology could also involve other alterations in the electrical activity of PFM motor units (MUs). Studying these alterations could provide novel insights into IC/BPS and help its clinical management. This study aimed to characterize PFM activity at the MU level in women with IC/BPS and pelvic floor myalgia using high-density surface electromyography (HD-sEMG). Signals were recorded from 15 patients and 15 healthy controls and decomposed into MU action potential (MUAP) spike trains. MUAP amplitude, firing rate, and magnitude-squared coherence between spike trains were compared across groups. Results showed that MUAPs had significantly lower amplitudes during contractions on the patients’ left PFM, and delta-band coherence was significantly higher at rest on their right PFM compared to controls. These findings suggest altered PFM tissue and neuromuscular control in women with IC/BPS and pelvic floor myalgia. Our results demonstrate that HD-sEMG can provide novel insights into IC/BPS-related PFM dysfunction and biomarkers that help identify subgroups of IC/BPS patients, which may aid their diagnosis and treatment.

## 1. Introduction

Chronic pelvic pain (CPP) is defined as a chronic or persistent pain perceived in structures related to the pelvis for at least 6 months [1] and is associated with modified pain processing mechanisms at both peripheral and central levels [2]. Its diagnosis and management pose significant challenges across various specialties due to its potential to present a broad spectrum of symptoms. Among them, interstitial cystitis/bladder pain syndrome (IC/BPS) is prevalent in 2% to 17.3% of the U.S. population [3]. It manifests as urinary urgency and frequency, and pain in the bladder and/or pelvis, without any identifiable cause [1]. Like other CPP-related disorders, the pelvic floor muscles (PFMs) play a significant role in the pathophysiology of IC/BPS. Approximately 85% of patients exhibit symptoms of myofascial pelvic pain and muscle overactivity [4]. The association between IC/BPS and PFM dysfunction can be explained by the close anatomical and neural connections between the brainstem projections from the PFM and those of the urethral and anal sphincters [5] and a phenomenon known as visceral–somatic convergence, which postulates that pain in a visceral organ can induce pain in adjacent, yet undamaged, tissues [6]. A consequence of these two factors is that a bombardment of painful stimuli originating from the bladder and other pelvic organs/structures to the spinal cord can initiate a wind-up effect that may result in heightened neural activity in the PFMs, causing their overactivation and vice versa [7]. Recent research has reinforced this connection between IC/BPS and PFM dysfunction, offering surface electromyography (sEMG)-based evidence of heightened PFM activation in patients with IC/BPS [8]. Specifically, this has been found in muscle areas of peak activity and in surrounding areas [9].

It is widely accepted that increased neural drive to the PFM is a common sign in CPP and particularly in IC/BPS. However, functional brain imaging studies have further revealed that the functional connectivity between certain brain regions involved in PFM control is altered in IC/BPS [10], which could result in the existence of other abnormal PFM motor control strategies beyond just increased neural drive to their motor units (MUs). This could imply a reorganization of muscle activity and inter-muscle coordination, along with the associated alterations in MU activity properties, as has been observed in other muscle pain conditions [11,12]. For PFM pain specifically, the existence of such kind of abnormal PFM motor control strategies was indirectly investigated in two recent studies that characterized the intermuscular coherence between the left and right PFM sides [13] and the intramuscular coherence within each side [14]. However, both analyses were performed from the signals recorded with sEMG, i.e., the summed extracellular postsynaptic potentials generated by the PFM MUs (MU action potentials, MUAPs). This approach suggests that coherence might have been underestimated, as MUAPs can function as high-pass filters for neural information [15]. Furthermore, neither study assessed other MU properties that can be affected in pain conditions, such as the MU firing rate or MUAP amplitude [12]. Their assessment necessitates identifying individual MU activity, which can be achieved by decomposing high-density surface electromyography (HD-sEMG) signals into individual MUAPs and MU spike trains (STs). Considering that previous findings have indicated abnormal PFM electrical activity and coherence at the sEMG level in this clinical context [8,13], as well as documented alterations in MU behavior in other muscles affected by pain [11,12], we hypothesized that MU activity characteristics are altered in IC/BPS

The aim of the present study was thus to assess alterations in PFM electrical activity in IC/BPS conditions through the characterization of individual MUAPs and STs obtained from decomposed HD-sEMG signals, and to infer specific information about IC/BPS pathophysiology in patients with PFM pain.

## 2. Materials and Methods

### 2.1. Sample Description

Thirty women were recruited in the study, of whom fifteen were patients diagnosed with IC/BPS according to the AUA/SUFU Guideline [16] and pelvic floor myalgia, and fifteen were healthy women with similar demographic characteristics (mean ± SD age in patients: 39.9 ± 15.1 years; in healthy women: 39.7 ± 14.2 years) and body mass index (patients: 27.5 ± 7.4 kg/m^2^; healthy women: 31.5 ± 11.3 kg/m^2^). On the other hand, the severity and impact of lower tract urinary symptoms were significantly higher in patients than in healthy women, as shown by their Interstitial Cystitis Symptom Index (9.9 ± 4.4 vs. 1.9 ± 2.4) and Interstitial Cystitis Problem Index (8.4 ± 3.6 vs. 0.3 ± 1.0), with a *p* < 0.001 in both cases (two-sample *t*-test). The study was conducted according to the guidelines of the Declaration of Helsinki at the Washington University School of Medicine, and its protocol was approved by its institutional review board and by that of the University of Houston. Informed consent was obtained from all subjects involved in the study. The inclusion criteria met by the patients were prior diagnosis of IC/BPS, bladder pain that increases with bladder filling or decreases after micturition, PFM pain upon palpation (pelvic floor myalgia), and older than 18 years old. The inclusion criteria for healthy women were the absence of PFM pain upon palpation or IC/BPS diagnosis. Exclusion criteria included pregnancy, endometriosis, or pelvic surgical histories in both groups.

### 2.2. Signal Recording and Decomposition

**Figure 1:**
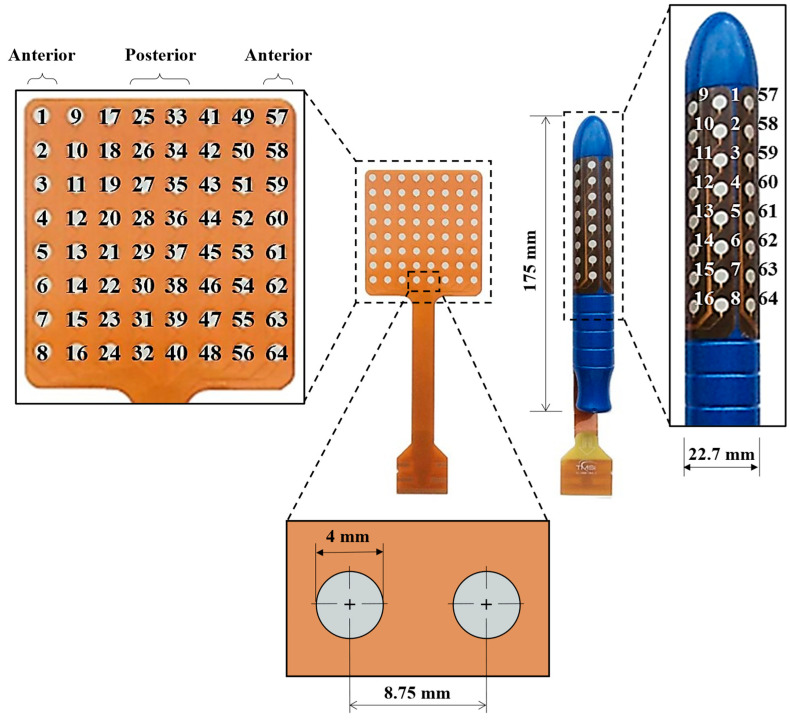
Dimensions of the grid of electrodes (numbered from 1 to 64) and intravaginal probe used for HD-sEMG detection.

**Figure 2:**
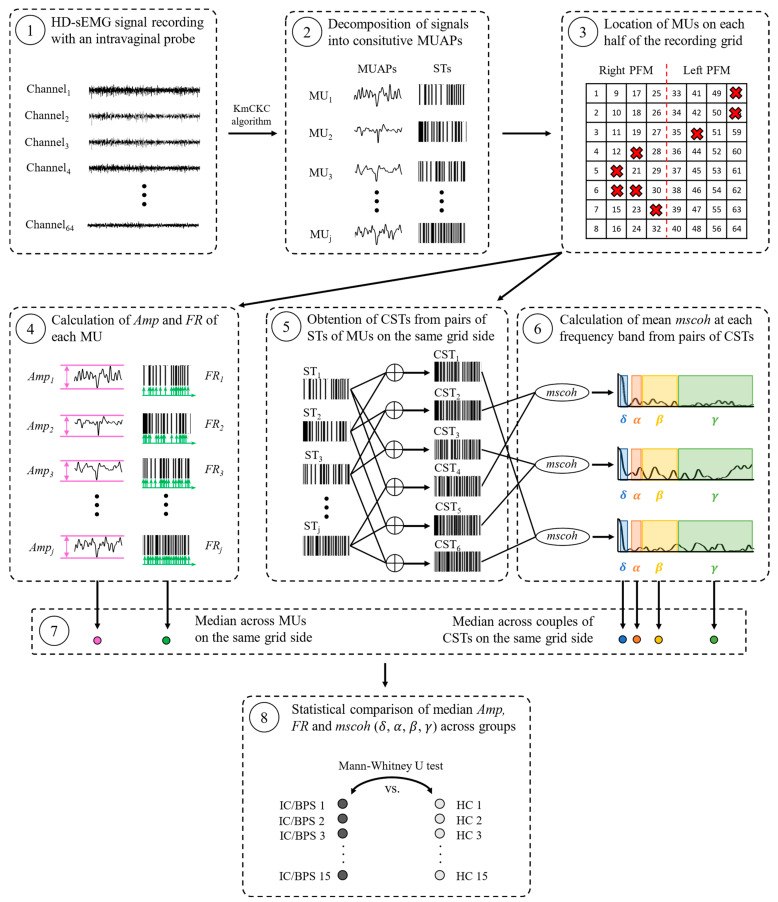
Steps performed to characterize MU properties from decomposed HD-sEMG signals and to compare them between IC/BPS patients and healthy controls. Red crosses on the HD-sEMG grid (3) show the MUs located on the right and left PFM.

Figure 1 depicts the grid of electrodes used to record HD-sEMG signals (left) and their arrangement on the plastic intravaginal probe (right). Figure 2 shows a schematic representation of the steps performed to assess the PFM electrical activity at the MU level, which were carried out separately for the activity recorded during PFM maximum voluntary contractions (MVC) and rest.

Patients remained in the dorsal lithotomy position, i.e., lying on their back with their hips and knees flexed and their legs elevated and spread apart, while their PFM electrical activity was recorded by HD-sEMG using a cylindrical intravaginal probe (length: 175 mm, diameter: 22.7 mm) that had a grid of 64 circular surface electrodes (diameter: 4 mm, interelectrode distance: 8.75 mm) attached to its surface.

The intravaginal probe was lubricated with a conductive gel for easier insertion into the vagina. It was positioned so that the first row of electrodes (closer to the probe tip) was closest to the innermost portion of the vagina, while the last row (closer to the probe base) was near the opening. Furthermore, the grid was oriented so that electrodes in the front columns (1–8 and 57–64) were closer to the urethra, and those in the middle columns (25–40) were closer to the anus, as shown in Figure 1.

To complete the setup, a fully soaked Velcro band was placed on the participants’ left wrist as a ground electrode, and a self-adhesive reference electrode was attached to the right thigh. PFM myoelectrical activity at rest was recorded for 60 s, and then the participant was asked to perform two 10 s-MVCs with a 10-s rest between consecutive contractions.

HD-sEMG signals were acquired at a sampling rate of 2048 Hz with a Refa 136-channel amplifier (TMSi, Enschede, The Netherlands) and offline filtered with two 2nd-order Butterworth filters: a bandpass filter (band-pass bandwidth: [10–500] Hz) and a notch filter at 60 Hz. Four 7 s- segments were annotated in the preprocessed signals for subsequent analysis. These were recorded during two PFM MVCs, the resting period before the first contraction, and the resting period between the two MVCs. They were then decomposed into constitutive MUAP trains using the K-means clustering convolution kernel compensation (KmCKC) algorithm [17] (Figure 2(2)). It estimates the MUAP pulse train of different MUs by (1) obtaining the global activity index of the multichannel recording; (2) collecting the time instants at which several MUs are simultaneously active, considered as the instants when the global activity index reaches the highest values; (3) clustering the observations of the multichannel recording at those time instants into different groups using the K-means clustering algorithm; (4) selecting the cluster with the largest number of observations and estimating the initial MUAP pulse train from the time instants associated with them, using the linear minimum square error method; and (5) applying a modified multi-step iterative convolution kernel compensation algorithm to update the estimated MUAP pulse trains. For each decomposed MU, the algorithm provides one MUAP template (with one waveform per channel, giving a total of 64 waveforms) by averaging the values of signal windows centered at the time instants of the spikes in the MUAP pulse train.

The position of each MU detected by the algorithm within the grid was designated as the channel at which the MUAP projection showed the greatest peak-to-peak amplitude (Figure 2(3)).

### 2.3. Characterization of MU Properties

Three MU characteristics were assessed from the MUAPs and STs provided by the KmCKC algorithm: MUAP amplitude (*Amp*), mean firing rate (*FR*), and magnitude-squared coherence (*mscoh*).

*Amp* was computed as the difference between the maximum and minimum values reached by the MUAP projection in the channel identified as the MU position, and *FR* was computed from STs as the mean of the inverse of the inter-spike interval between consecutive MU discharges (Figure 2(4)). *Amp* and *FR* were calculated for every MU identified by the algorithm and their median values were obtained separately across the MUs located on the left and right halves of the grid (Figure 2(7)), considering the innervation of each PFM is independent of that of the other side [18] and this could imply substantial differences in their MU activity properties.

Cumulative spike trains (CSTs) were obtained from the STs of each possible pair of MUs located on the same half of the HD-sEMG grid (left or right) (Figure 2(5)). *Mscoh* was then computed for the impulse trains of each possible pair of CSTs that met the condition of having been constituted from four different MUs (Figure 2(6)). For example, if the algorithm detected 4 MUs on a given PFM side, 6 CSTs were obtained from their STs: CST_1_(ST_MU1_⨁ST_MU2_), CST_2_(ST_MU1_⨁ST_MU3_), CST_3_(ST_MU1_⨁ST_MU4_), CST_4_(ST_MU2_⨁ST_MU3_), CST_5_(ST_MU2_⨁ST_MU4_), and CST_6_(ST_MU3_⨁ST_MU4_). *mscoh* was computed between the CSTs of the following pairs: {CST_1_, CST_6_}, {CST_2_, CST_5_}, and {CST_3_, CST_4_}. Hence, if x[n] and y[n] are the time series associated with two CSTs, their *mscoh* was computed to characterize the similarities in their spectral content at each frequency as follows:

(1) $$mscoh\left(f\right)=\frac{{\left|C_{xy}\left(f\right)\right|}^{2}}{C_{xx}\left(f\right)\cdot C_{yy}(f)}$$

where $C_{xy}\left(f\right)$ is their cross-spectral density function and $C_{xx}\left(f\right)$ and $C_{yy}(f)$ their autospectral density functions at the frequency f. In the present study, $C_{xy}\left(f\right)$, $C_{xx}\left(f\right)$, and $C_{yy}(f)$ were calculated with non-overlapping windows of 1 s [19].

The *mscoh* of every CSTs pair was summarized by the average of its values across the frequencies of four bandwidths: delta ($mscoh_{\delta }$: 1–5 Hz), alpha ($mscoh_{\alpha }$: 8–12 Hz), beta ($mscoh_{\beta }$: 13–30 Hz) and gamma ($mscoh_{\gamma }$: 31–70 Hz). The median value of $mscoh_{\delta }$, $mscoh_{\alpha }$, $mscoh_{\beta }$, and $mscoh_{\gamma }$ across the CSTs pairs on the same half of the grid was then calculated (Figure 2(7)).

*Amp*, *FR*, and *mscoh* were analyzed separately for each PFM side during (1) the resting phase before the first MVC and (2) the second MVC conducted by the patients. Segment (1) was chosen to characterize PFM resting activity, considering that the resting period between consecutive contractions might not accurately reflect PFM tonic activity in daily life, especially for patients, as the muscle could attain a higher relaxation level post-MVC [20]. Segment (2) was selected to assess PFM MVC, noting that many participants initially struggled to properly complete the PFM MVC maneuver due to unfamiliarity on their first attempt. If the decomposition algorithm failed to detect at least 4 MUs in either segment (1) or (2), the resting interval between the two contractions and the first contraction executed by the patient were analyzed instead.

### 2.4. Statistical Analysis

Statistically significant differences between the patients’ and the healthy controls’ *Amp*, *FR*, and *mscoh* values at each PFM activation state (MVC or rest) and side (left or right) were assessed by the Mann–Whitney U test at a 5% confidence level (Figure 2(8)). In the case of *mscoh*, it was assumed that it showed different values between patients and healthy controls if any of its mean values at the four frequency bands (delta, alpha, beta, gamma) were statistically significantly different across groups. After applying the Bonferroni correction, a 1.25% confidence level was adopted.

## 3. Results

### 3.1. Decomposition Algorithm Performance

Table 1 gives a summary of the decomposition algorithm performance. The data reveals that more MUs were identified on the right side of patients than on their left, with this difference being statistically significant only during PFM rest (9.8 ± 6.5 vs. 3.5 ± 3.9; *p*-value = 0.029). In contrast, MU detection was similar across both PFM sides in healthy controls. Notably, the number of MUs detected on the patients’ right PFM side was significantly higher than that in healthy controls, but this was only significant during muscle rest (9.8 ± 6.5 vs. 5.3 ± 6.6; *p*-value = 0.023). Table 1 also shows that the IC/BPS group included more analyzable cases than the HC group when their right PFM were compared (13 vs. 8 during MVC and 14 vs. 8 at rest), and that the number of MUs and CSTs analyzed was significantly lower on the patients’ left side than on healthy controls at PFM rest (No. MUs: 6.6 ± 3.2 vs. 11.4 ± 5.2; No. CSTs: 23.0 ± 23.8 vs. 70.6 ± 65.8; *p*-value = 0.027).

### 3.2. MUAP Amplitude and Firing Rate

**Figure 3:**
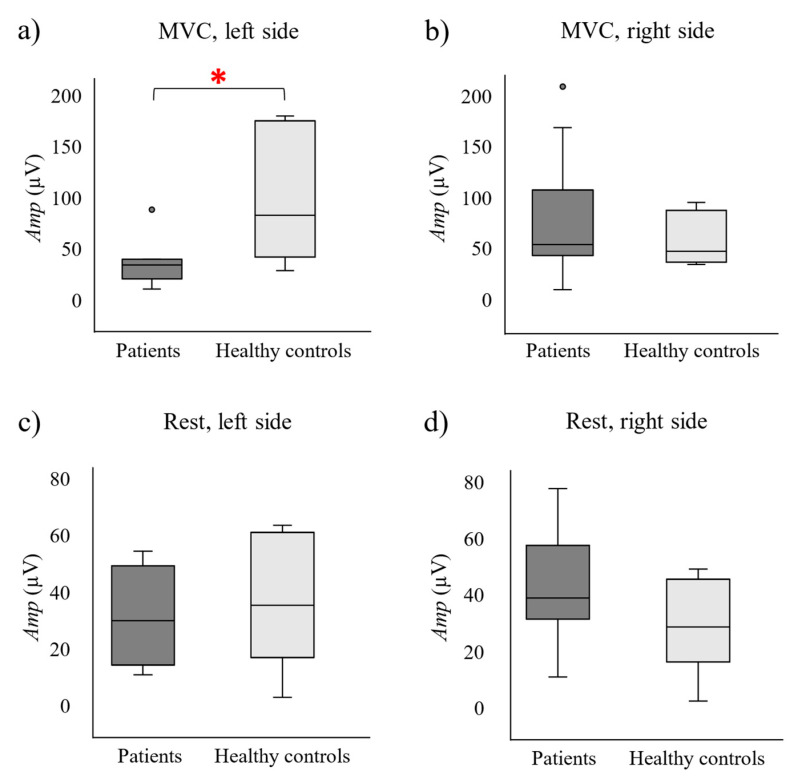
Motor unit action potential amplitude (*Amp*) on the PFM left and right sides in patients (dark boxes) and healthy controls (light boxes) during pelvic floor muscles’ maximum voluntary contractions (MVC; **a**,**b**) and at rest (**c**,**d**). (*): statistically significant difference.

**Figure 4:**
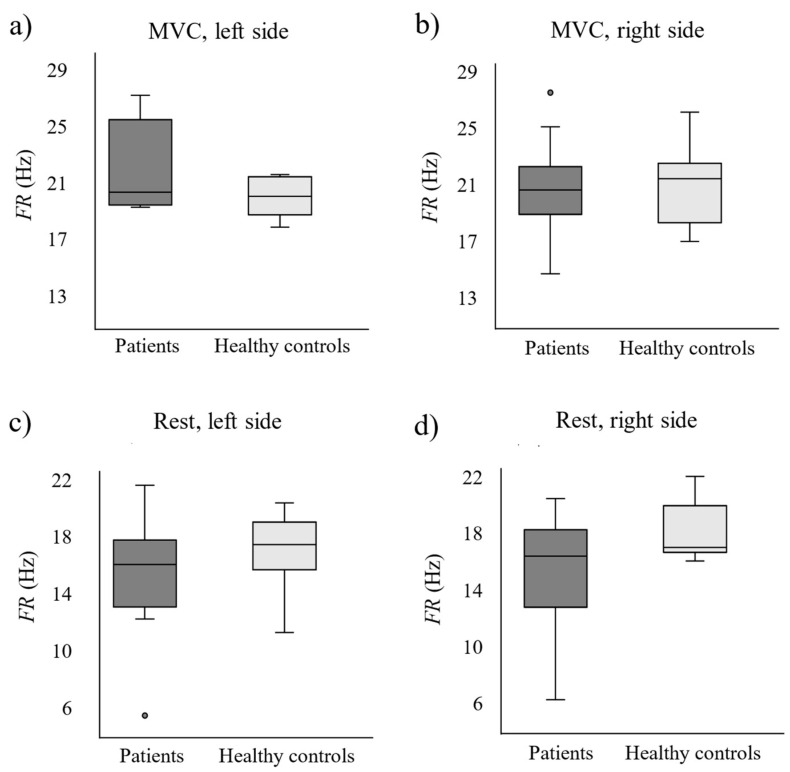
Motor unit action potential firing rate (*FR*) on the PFM left and right sides in patients (dark boxes) and healthy controls (light boxes) during pelvic floor muscles maximum voluntary contractions (MVC; **a**,**b**) and at rest (**c**,**d**).

Figure 3 and Figure 4 show the box–whisker plots of the patients’ (dark grey boxes) and healthy controls’ (light grey boxes) *Amp* and *FR* on their left and right PFM sides during PFM MVC and rest. Statistically significant differences between patients and healthy controls are highlighted with an asterisk.

As shown in Figure 3, *Amp* had significantly lower values on the patients’ left side during PFM MVC (71.3 ± 53.1 μV) than on the healthy controls’ (112.5 ± 80.8 μV, *p*-value = 0.030, Figure 3a). On the other hand, its values were not significantly different across groups on that same PFM side at muscle rest (*p*-value = 0.384) nor on the right side on either PFM activity state (*p*-value_MVC_ = 0.304; *p*-value_rest_ = 0.098). As for *FR,* Figure 4 shows a trend of lower values in patients than in healthy women at PFM rest, especially on their right PFM, although differences were not statistically significant (Right: *p*-value = 0.090; Left: *p*-value = 0.411). There were no statistically significant differences in *FR* values across groups during MVCs either (right: *p*-value = 0.765; left: *p*-value = 0.215).

### 3.3. Coherence Between MU Spike Trains

**Figure 5:**
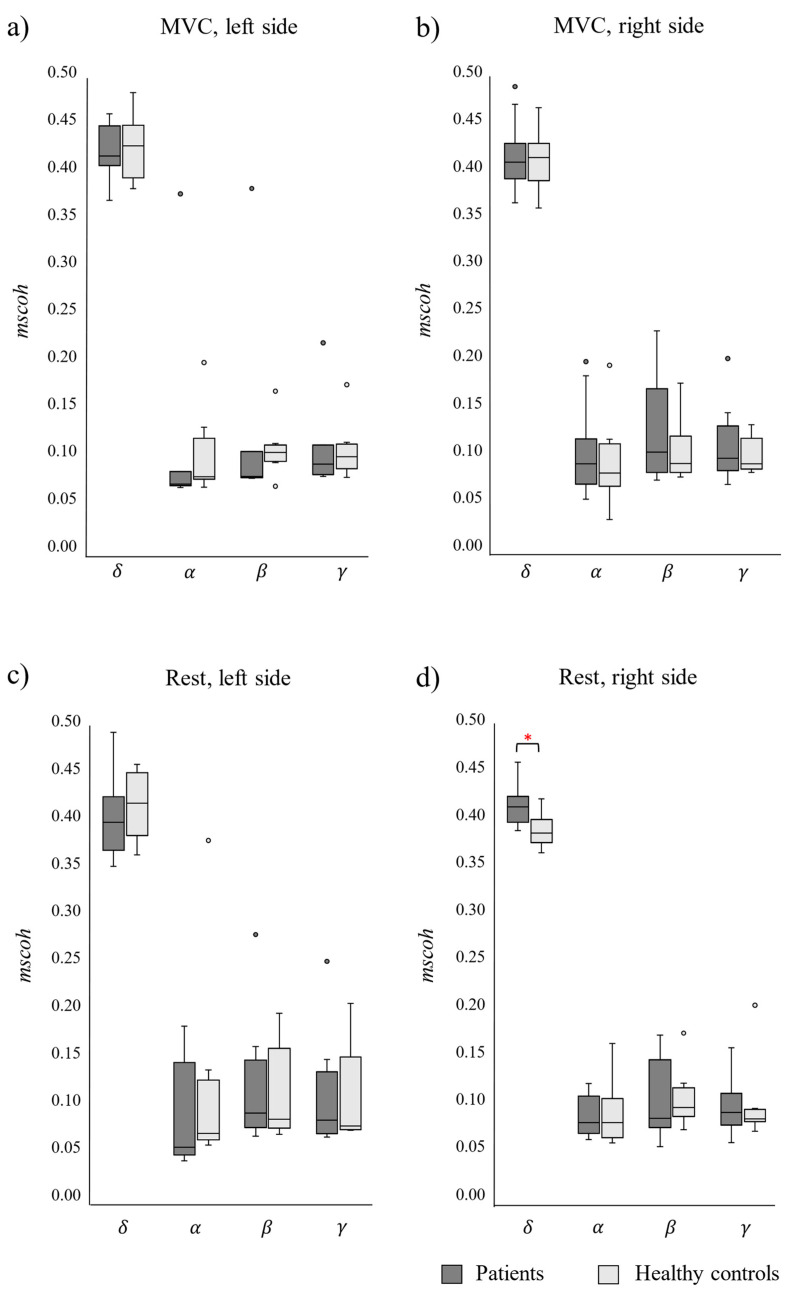
Magnitude-squared coherence (*mscoh*) on the left and right PFM in patients (light boxes) and healthy controls (dark boxes) during pelvic floor muscles maximum voluntary contractions (MVC; **a**,**b**) and at rest (**c**,**d**). (*): statistically significant difference.

Figure 5 shows the box–whisker plots of the patients’ and healthy controls’ *mscoh* on their left and right PFM sides during PFM MVC and rest. Values in the delta (δ), alpha (α), beta (β), and gamma (γ) bands are represented with different boxes and significant differences between patients and healthy controls are highlighted with an asterisk.

Delta-band *mscoh* values on the right side at PFM rest (Figure 5d) were significantly higher in patients (0.42 ± 0.02) than in healthy controls (0.39 ± 0.02, *p*-value = 0.007). On the other hand, there were no statistically significant differences across groups for this same PFM side and state of muscle activation in the other frequency bands (α: *p*-value = 0.345; β: *p*-value = 0.976; γ: *p*-value = 0.853). There were no statistically significant differences on the left PFM *mscoh* values across groups in any band either (Figure 5a,c).

## 4. Discussion

The present study assessed changes in the PFM activity at the MU level in women with IC/BPS and PFM pain using HD-sEMG recordings. In this analysis, three parameters frequently assessed in research on MU activity reorganization in muscle pain conditions were examined: MUAP amplitude (*Amp*), mean firing rate (*FR*) of MUs, and coherence (*mscoh*) between pairs of CSTs. Its results showed that MU activity is altered in IC/BPS patients with pelvic floor myalgia. In particular, they demonstrated that MUAPs on their left PFM show significantly decreased amplitudes during PFM MVCs, and delta-band coherence between spike trains fired at PFM rest is significantly higher on the right PFM than in healthy women. These results are discussed in more depth in the subsections below.

### 4.1. Effect of IC/BPS and PFM Pain on the PFM MU Properties: MUAP Amplitude and Firing Rate

According to the results of the present study, MUAP amplitude had significantly lower values on the patients’ left side at PFM rest, which could be a sign of muscle tissue changes associated with PFM myalgia associated with IC/BPS such as muscle fiber atrophy, loss and/or splitting [21], and none of the two PFM sides showed significantly different MU mean firing rates across groups. These findings may appear inconsistent with previous research in the field, as earlier studies have reported no changes in MUAP amplitude in muscle pain conditions [22,23], and indicated that high-threshold MUs tend to increase their firing rate during pain, while low-threshold MUs either decrease it or maintain it [11,23]. However, they focused on experimentally induced muscle pain, whereas other research examining clinical muscle pain has reported less uniform changes in MUAP amplitude and MU firing rates [12]. This suggests that the alterations in muscle activity observed in experimentally induced pain may not fully replicate those occurring in chronic pain, thereby explaining the discrepancies between the results of our study, which examined chronic muscle pain, and those in previous studies [11,22,23]. Additionally, unlike in the studies by Hodges et al. [11] and Martinez–Valdes et al. [23], low and high-threshold MUs were analyzed collectively in the present study. As a result, opposing changes in firing rates could have neutralized each other, leading to no overall change in the mean firing rate of MUs.

### 4.2. Effect of IC/BPS on the PFM MU Properties: Coherence Between MU Spike Trains

According to a recent review [12], only two studies had previously evaluated the intramuscular coherence between STs or CSTs in muscle pain conditions, and pain was experimentally induced rather than clinical pain in both cases [24,25]. One of them ([25]) evaluated changes in the MU activity properties of the upper trapezius muscle in pain-free vs. pain conditions and reported an increase of its intramuscular coherence peak in the delta band when its cranial and caudal regions were separately assessed in pain conditions. Our study’s findings align with these observations, demonstrating that delta-band intramuscular coherence on the right PFM of patients is significantly greater than that in healthy women during muscle rest, as shown by significantly higher *mscoh* values. As for the physiological implication of this result, increased coherence in the delta band indicates a heightened common drive or instantaneous co-modulation of MU firing rates in IC/BPS conditions [26]. This phenomenon could substantially affect the stability of PFM resting activity, which is known to be reduced in CPP [27]. Notably, higher coherence in this bandwidth has been linked to decreased steadiness in muscle force [19].

Dideriksen et al. [25] and Yavuz et al. [24] also reported significant changes in the muscle alpha-band coherence [24,25] and beta-band coherence [24] during experimental pain; however, we did not identify significant differences at these frequency bands. This discrepancy could stem from two main factors: (1) the difference in muscles examined (trapezius, tibialis anterior, and abductor digiti minimi vs. PFM) and (2) the nature of the pain (experimental vs. clinical). For the first factor presented, Yavuz et al. indicated that pain-related coherence changes might vary based on specific muscle characteristics like the corticospinal projections to the muscle’s motor neurons. Regarding the second factor, as previously mentioned, the impact of pain on MU properties, particularly coherence, is believed to differ between experimentally induced and clinical pain [12].

It should be mentioned that a previous study evaluated PFM intramuscular coupling in conditions of clinical pain, although at a more global level from sEMG signals, and showed that their cross-correlation, from which *mscoh* definition derives [28], was significantly lower in CPP patients with deep dyspareunia than in their healthy counterparts [14]. However, cross-correlation was not evaluated in narrow frequency bandwidths (delta, alpha, beta, gamma) but in a single wide bandwidth ([30–450] Hz), and MVC and resting activity segments were assessed jointly, which hampers the possibility of comparing its results with those of the present study.

The conclusions in [14] have recently been supported by a second study that assessed cross-correlation and other coupling metrics from the same database before and after treating patients with BoNT/A injections [29]. It evaluated PFM electrical coupling in a more reduced bandwidth ([0–10] Hz) of rectified and low-pass filtered sEMG signals, and its results showed that cross-correlation was significantly lower in patients during MVC than in healthy controls rather than higher, as shown by our results. Coherence estimates obtained from sEMG and MUs are only correlated for frequencies higher than 5 Hz and at low contraction levels [15], implying that only beta, alpha, and gamma band coherence at PFM rest could have been reliably compared across studies if resting activity had also been assessed in [29]. Despite this, it must be highlighted that its results also showed that PFM intramuscular coherence was altered even after treatment with BoNT/A, which could be explained by the inability of the nervous system to restore its original neuromuscular control strategies even after pain resolution, among other causes, and thus be related to the recurrence of painful symptoms months after treatment. Given that coherence analysis at the MU level provides a more reliable vision of neuromotor control strategies than sEMG recordings, further efforts should be made to contrast this hypothesis by assessing changes in the delta-band intramuscular coherence after IC/BPS treatment and the correlation between its value, the intensity of painful symptoms (before and after treatment) and their recurrence months after treatment.

### 4.3. Impact of IC/BPS on the Left and Right PFM Sides

The decomposition algorithm detected more MUs on the patients’ right PFM side than on their left side, while the detection was more similar across sides in the case of healthy controls. Furthermore, MUAP amplitude was significantly different across the groups’ left side but not across their right side, which would be explained by significantly lower MUAP amplitudes on the patients’ left PFM with respect to their right PFM, as obtained in subsequent comparisons. Intramuscular coherence was also significantly higher in patients than in healthy controls but only on the right side of their PFMs. Additional intragroup analyses showed that its values were significantly different across the healthy controls’ PFM sides at PFM rest, while it was not different across the patients’ PFM sides, thus implying that the common comodulating drive that the MUs receive is more similar across PFM sides in IC/BPS. This indicates that CPP may exert varying influences on each side of the PFM. This was also observed in previous studies, as Knowles and Cohen [30] showed that tenderness upon palpation is more common on the left side than on the right side in chronic anal pain associated with PFM tension or spasms. Another study on CPP associated with dyspareunia reported that the left PFM side was the only or the most painful in most of the participants [14] An additional analysis performed on the same sample of patients as in [14] revealed significantly altered PFM electrical coupling within their most painful side but not within their opposite side [29], and a recent study showed that changes in the PFM resting activity after treatment with botulinum toxin injections were significantly related with changes in the vulvar sensitivity on the right side but not on the left side [31]. The underlying cause of this notable disparity between the two sides of the PFMs remains unclear. However, it has been speculated that factors like uneven load distribution on the hips and PFMs [32], or the nature of delivery and pelvic trauma, whether obstetric or non-obstetric [33], may contribute to this variation. Future studies should thus assess in more depth whether CPP has a truly distinct impact on each PFM side, the clinical or electrophysiological origin of this imbalance, and why the PFM side that is more affected by the pathology varies depending on the feature assessed or the main CPP manifestation/symptom of the patient (IC/BPS, dyspareunia, anal pain, etc.). If a variable influence of CPP on each PFM side was confirmed, further efforts should also be made to determine whether this has any clinical implications in the prognosis or treatment of CPP, and specifically IC/BPS, or whether it is associated with greater risk or severity of lower urinary tract or PFM spasm symptoms such as urinary urgency, urinary frequency, or constipation.

### 4.4. HD-sEMG Compared to Other PFM Assessment Techniques and Its Role in IC/BPS Diagnosis and Treatment

HD-sEMG is an objective measurement technique that offers advantages over other techniques currently available for assessing PFM in pelvic health conditions, particularly, IC/BPS. Most measure PFM tone and contractile strength, including subjective techniques such as digital palpation and objective techniques such as dynamometry and perineometry [34]. While these PFM features cannot be measured directly with HD-sEMG, they can be assessed indirectly by quantifying PFM electrical activity at rest and during voluntary contractions. Compared to digital palpation, the main advantage of HD-sEMG lies in its ability to provide objective measures that do not rely on the subjective perception of the physician who performs the pelvic physical examination. Compared to dynamometry and perineometry, HD-sEMG allows assessing individual muscles or PFM sides rather than providing general measures for the whole muscle [34], and obtaining more detailed and specific information about PFM neuromotor control strategies that can be relevant for getting new insights into IC/BPS pathophysiology, as seen in the present study. On the other hand, information about the passive component of PFM tone cannot be obtained from HD-sEMG, and the interpretation of signal features obtained with advanced signal processing methods can be challenging for non-engineering professionals, which might limit the use of HD-sEMG in clinical settings.

Compared to “traditional” sEMG recordings, HD-sEMG allows monitoring of PFM myoelectrical activity at multiple sites with a high spatial resolution. This is a significant advantage, as PFM myoelectrical activity is not heterogeneous across different muscle areas [8,9,35] and the activity of abnormal PFM areas may be masked in global sEMG recordings. In addition, decomposition algorithms can be applied to HD-sEMG signals to separate overlapping MUAPs, as in the present study. This allows studying PFM electrical activation at the MU level, which prevents results from being biased by factors that affect “traditional” sEMG recordings, such as MUAP cancellation, muscle location and morphology, and low spatial filtering through conductive tissue [36]. On the other hand, the volume of data generated with HD-sEMG is much larger than with traditional sEMG, which implies higher storage and computational costs. Furthermore, the relative location of the electrodes to the different PFM bundles is variable across subjects due to PFM morphology variability [37]; therefore, findings at a specific electrode cannot be reliably associated with a specific muscle area or layer. A recent study that attempted to solve this limitation presented a novel HD-sEMG device intravaginal probe that consisted of a low-modulus inflatable airbag and a stretchable array of electrodes spatially distributed so that they made contact with the muscle belly of the main deep and superficial PFMs [35]. However, this device requires some imaging techniques to confirm the location of the different muscles in a specific subject.

As regards the role of HD-sEMG in IC/BPS clinical management, a recently published study indicated that there is a need to identify relevant subgroups among IC/BPS patients, who may present a broad spectrum of pathophysiological phenotypes, to provide more effective treatment that considers the patient’s clinical profile [38]. However, defining such subgroups requires identifying relevant novel biomarkers that may be used in conjunction with already existing IC/BPS biomarkers. The present study has shown that PFM myoelectrical activity features such as MUAP amplitude or delta-band intramuscular coherence are altered in patients with IC/BPS and pelvic floor myalgia, which suggests that HD-sEMG could play a relevant role in identifying specific biomarkers that help define subgroups among IC/BPS patients to achieve a more effective treatment.

### 4.5. Limitations

The main limitation of the study was the small sample size. Although it was similar to that of recent studies that also assessed MU activity in muscle pain [11,23], the ability to generalize the results to a larger population may be limited due to the low number of participants included in the analyses. Furthermore, this and the limited number of participants in which at least four MUs were detected on both PFM sides prevented us from also assessing PFM intermuscular coherence and thus from contrasting our results with those reported by Houston et al. [13]. Further efforts should thus be made to increase the sample size in future studies. This can be challenging, as the center’s patient load limits the ability to recruit patients who meet the study’s inclusion criteria. This could be solved by designing multicenter studies, which would also reduce the bias in the results associated with carrying out the study in a single center and by the same evaluators.

Regarding the asymmetry of the results between the two muscle sides, while they could have a pathophysiological origin or implication, placing the reference electrode on only one side could also have affected the signals detected from the left and right PFMs differently, as described in [39]. It would thus be advisable to use two reference electrodes, one per side, or alternate the location of a single electrode between left and right tights across different participants in future studies to corroborate that the asymmetry of the results between the two sides is not due to a limitation in the recording protocol.

Finally, further efforts should also be made to include concurrent measures of intravaginal pressure to label MUs according to their recruitment threshold and thus to study separately the changes that low and high-threshold MUs experience in IC/BPS, as current literature supports that they are not uniform across the motor neuron pool [11]. Other measures that should be included in future studies to provide a more comprehensive picture of PFM dysfunction in IC/BPS conditions are PFM stiffness, elasticity, and tone.

## 5. Conclusions

This is the first study to assess alterations in the PFM myoelectrical activity at the MU level in IC/BPS using HD-sEMG. While the mean firing rate was not altered, patients showed lower MUAP amplitude during MVC and higher delta-band intramuscular coherence at PFM rest. These findings support the existence of changes in the muscle tissue and altered neuromuscular strategies related to PFM control in IC/BPS conditions, which is one of the hallmarks of muscle pain and which could be difficult to revert as they were developed over a long time. These alterations were only observed on one PFM side, which adds the present study to the current literature that supports a distinct degree of clinical/electrophysiological alterations across PFM sides in CPP.

The results of this study point to the potential value that the assessment of PFM myoelectrical activity at the MU level using HD-sEMG recordings could have to evaluate IC/BPS severity or impact from a perspective that would be complementary to the one offered by the patient’s symptoms description. It could be used to assess the magnitude of altered PFM neural control strategies or PFM tissue modifications, which would be associated with the chronicity of IC/BPS symptoms. It could also provide new insights into the effect of IC/BPS therapies, which is highly variable among patients and even completely unsuccessful for some of them.

## Figures and Tables

**Table 1:** Summary of the total and mean number of MUs detected on each PFM side and activation state in IC/BPS patients (IC/BPS) and healthy controls (HC).

	MVC				Rest			
	Left		Right		Left		Right	
	IC/BPS	HC	IC/BPS	HC	IC/BPS	HC	IC/BPS	HC
Total no. MUs detected	74	86	144	86	53	91	157	89
No. MUs detected (mean ± SD)	4.8 ± 5.5	5.9 ± 6.6	9.7 ± 6.5	6.7 ± 6.7	3.5 ± 3.9 ^#^	4.7 ± 7.1	9.8 ± 6.5 *^#^	5.3 ± 6.6 *
No. subjects with ≥4 MUs (%)	7 (47%)	8 (53%)	13 (87%)	8 (53%)	8 (53%)	8 (53%)	14 (93%)	8 (53%)
No. MUs (mean ± SD)	10.6 ± 2.7	10.8 ± 5.4	11.1 ± 5.9	11.4 ± 6.0	6.6 ± 3.2 *	11.4 ± 5.2 *	11.2 ± 5.6	11.1 ± 5.5
No. CSTs (mean ± SD)	53.7 ± 28.2	65.4 ± 57.9	71.8 ± 64.6	74.5 ± 63.3	23.0 ± 23.8 *	70.6 ± 65.8 *	72.0 ± 66.8	69.8 ± 65.0

SD: standard deviation. (*): *p*-value (IC/BPS vs. HC) < 0.05. (#): *p*-value (left vs. right) < 0.05.

## Data Availability

Data supporting the results presented in the manuscript are available upon reasonable request.
